# A novel double-mAb sandwich ELISA utilizing broad-spectrum neutralizing antibodies as an *in vitro* alternative to the rabies vaccine NIH potency assay

**DOI:** 10.3389/fimmu.2026.1960986

**Published:** 2026-10-07

**Authors:** Meina Cai, Xi Wu, Leitai Shi, Jincheng Tong, Chenchen He, Jianhui Nie, Yi Yang, Jinkai Ren, Yuhua Li, Qunying Mao, Youchun Wang, Shouchun Cao, Weijin Huang

**Affiliations:** 1 Division of HIV/AIDS and Sexually Transmitted Virus Vaccines, Institute for Biological Product Control, National Institutes for Drug Control (NIDC), Beijing, China; 2 Changping Laboratory, Beijing, China; 3 Department of Arboviral Vaccine, National Institutes for Drug Control (NIDC), Beijing, China; 4 Chinese Academy of Medical Sciences & Peking Union Medical College, Beijing, China; 5 Institute of Medical Biology, Chinese Academy of Medical Science & Peking Union Medical College, Kunming, Yunnan, China; 6 Shenyang Pharmaceutical University, Shenyang, China; 7 Biocytogen Pharmaceuticals Co., Ltd., Beijing, China; 8 Beijing Wantai Biological Pharmacy Enterprise Co., Ltd., Beijing, China

**Keywords:** ELISA, *in vitro* alternative method, monoclonal antibodies, pseudovirus neutralization, rabies virus

## Abstract

**Background:**

Rabies remains a devastating zoonotic disease, with prevention measures relying heavily on post-exposure prophylaxis (PEP). Evaluating rabies vaccine potency traditionally relies on the *in vivo* National Institutes of Health (NIH) mouse challenge assay, which is limited by long testing cycles, low reproducibility, and high animal consumption. Developing broad-spectrum monoclonal antibodies (mAbs) and alternative *in vitro* assays is essential to advance rabies prevention and improve vaccine quality control.

**Methods:**

In this study, we characterized three human-mouse chimeric mAbs (05-2F10, 06-2A12, and 07-4D8-1C1) with broad-spectrum neutralizing activity. Leveraging *in vitro* pseudovirus neutralization assays, we evaluated candidate mAbs for high neutralizing capacity and low sensitivity to variation to select optimal pairs for assay development. Using these selected mAbs, a double-antibody sandwich ELISA was established to quantify glycoprotein (G) antigen content in human rabies vaccines.

**Results:**

The three chimeric mAbs exhibited exceptional *in vitro* neutralization potency and breadth. The established double-antibody sandwich ELISA successfully quantified G antigen content in human rabies vaccines. Importantly, a high correlation was demonstrated between the antigen content quantified by this ELISA and the potency data generated via the traditional *in vivo* NIH mouse challenge test.

**Conclusion:**

Collectively, these findings provide a robust technical framework for replacing the traditional NIH potency assay with a reliable *in vitro* method, fully aligning with the 3Rs principles of animal welfare.

## 1 Introduction

Rabies is a devastating zoonotic infectious disease caused by the rabies virus (RABV), a member of the *Lyssavirus* genus within the *Rhabdoviridae* family (1–3). The ~12 kb single-stranded RNA genome of RABV encodes five structural proteins: nucleoprotein (N), phosphoprotein (P), matrix protein (M), glycoprotein (G), and RNA polymerase large protein (L) (4). The surface-anchored G protein is the sole viral envelope component responsible for mediating viral entry by binding to host target cell receptors. It serves as the primary protective antigen capable of inducing virus-neutralizing antibodies (VNAs), making it the critical target for host immune responses and vaccine design (5–8).

Given that no specific curative treatments exist once clinical symptoms manifest, timely post-exposure prophylaxis (PEP) remains the only life-saving intervention. Standard PEP protocols include immediate wound cleansing, a series of rabies vaccinations, and the administration of passive rabies immunoglobulin (RIG) for Category II (if immunocompromised) and Category III exposures. Clinical RIG formulations predominantly comprise human RIG (hRIG) or equine RIG (eRIG). However, their global implementation is hampered by exorbitant manufacturing costs, chronic supply shortages, and potential batch-dependent safety risks. To circumvent these limitations, monoclonal antibodies (mAbs) have emerged as highly viable alternatives due to their high specificity, consistency, and potential for scalable, low-cost mass production. Notable examples include Rabishield, launched in 2017 (9–12); the RVC-20/RVC-58 antibody cocktail discovered by De Benedictis et al. (13); SYN023 reported by Chao et al. (14); and the NM57S/NC08 combination developed by North China Pharmaceutical Corporation—all of which have demonstrated potent neutralizing capacity and robust *in vivo* protection in animal models.

Recognizing these constraints, international consensus emphasizes that ELISAs targeting the native conformation of the G protein represent the most promising alternative to the traditional NIH assay. Although several ELISA frameworks have been established globally, commercially available kits in China remain extremely limited. Furthermore, existing domestic assays frequently suffer from poor strain compatibility—failing to reliably quantify G protein content across diverse vaccine strains (such as PM or CTN-1V) or different host cell substrates. Many also rely on antibodies targeting linear epitopes, resulting in poor correlation with *in vivo* biological potency, particularly when evaluating heat-stressed or degraded vaccines.

To address these critical gaps, we developed and characterized a panel of broad-spectrum human-mouse chimeric mAbs targeting highly conserved neutralizing epitopes (Site I and Site IIa) on the native RABV G protein. Leveraging their distinct non-competing specificities and exceptional neutralization breadth across 58 street isolates and 5 major vaccine strains, we established a double-antibody sandwich ELISA. This assay overcomes strain-specific bias, providing a universally applicable, highly robust platform that demonstrates an exceptional correlation (*R*^2^ = 0.91) with the traditional *in vivo* NIH potency assay, thereby offering a reliable 3Rs-compliant alternative for rabies vaccine quality control.

## 2 Results

### 2.1 mAb selection and characterization

To generate mAbs with potent neutralizing capabilities against RABV, BALB/c mice were immunized with a mammalian expression plasmid encoding the G gene of the CVS-N2c strain. Fusion and flow cytometry screening yielded eight positive hybridoma clones displaying high binding affinity for the native G protein. Variable regions were grafted onto human IgG1/kappa constant frameworks to generate human-mouse chimeric mAbs (05-2F10, 06-2A12, 07-4D8-1C1, 15-2G4-1F2, 05-2C12, 09-1G11, 09-3B10-1G11, and 09-2F8).

The neutralizing breadth and potency of all eight chimeric mAbs were systematically benchmarked against a panel of 58 regional street virus isolates (**Figure 1a**) and 5 standard vaccine strains (aGV, CTN-1V, PV, Flury-LEP, and PM) (**Figure 1b**) using high-throughput pseudovirus neutralization assays.

**Figure 1:**
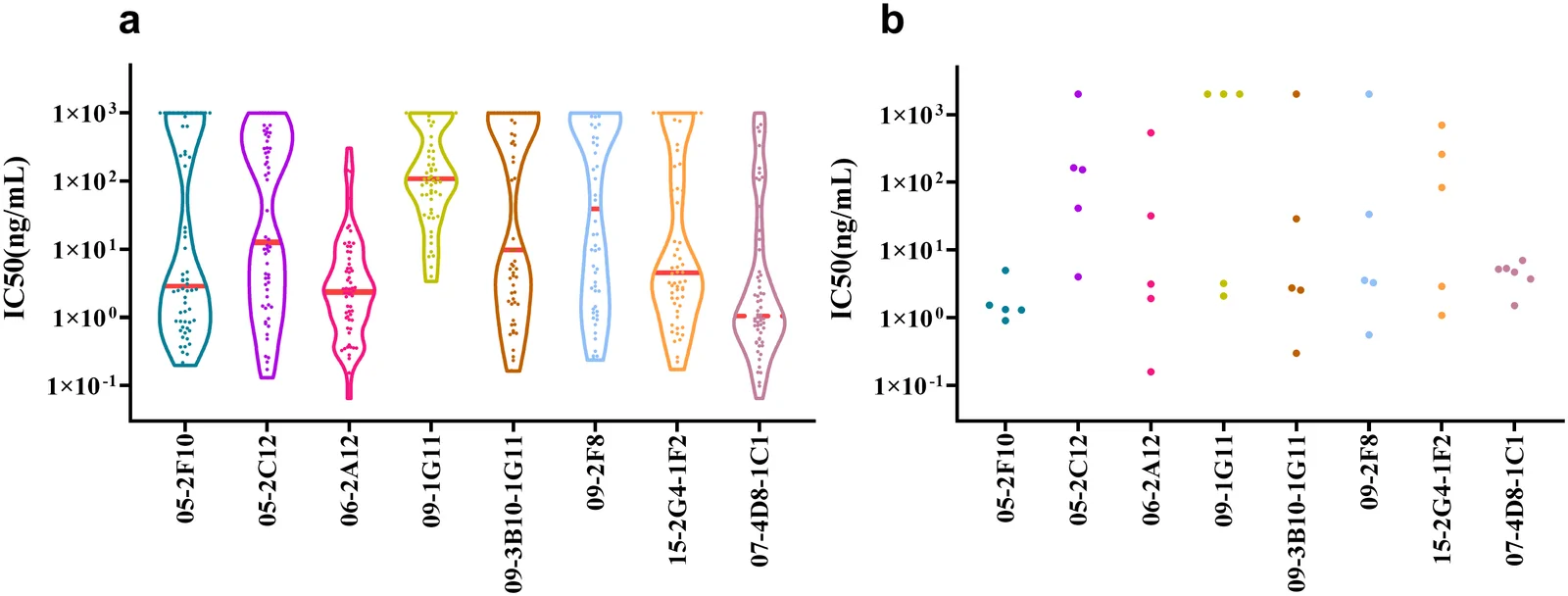
Neutralization activity of mAbs against rabies viruses. Vaccine strains and selected street virus strains were prepared into pseudoviruses, and the neutralizing activity of monoclonal antibodies was evaluated using *in vitro* neutralizing experiments based on pseudoviruses. Panel **(a)** shows the neutralizing activity of the mAbs against the street virus strains, with the red line representing the median IC50 value. Panel **(b)** shows the neutralizing activity of the mAbs against the vaccine strain pseudovirus.

Among the candidates, mAb 06-2A12 exhibited the highest overall neutralizing activity against street isolates, neutralizing all 58 strains with IC50 values ranging from 0.065 to 303.83 ng/mL (median IC50: 2.43 ng/mL) (**Figure 1a**). MAbs 07-4D8-1C1 and 05-2F10 also showed superior performance, neutralizing 48 street isolates with IC50 values below 1000 ng/mL (median IC50: 0.96 ng/mL and 2.18 ng/mL, respectively) (**Figure 1a**). Crucially, when cross-evaluated against the 5 major vaccine strains, mAbs 05-2F10 and 07-4D8-1C1 displayed exceptional and uniform potency across all strains, with IC50 values spanning 0.68–5.02 ng/mL and 1.52–7.05 ng/mL, respectively, followed by 06-2A12 (IC50: 0.16–539 ng/mL) (**Figure 1b**).

In contrast, the remaining candidates were excluded from further sandwich ELISA development due to insufficient breadth or potency. Specifically, mAbs 05-2C12, 09-3B10-1G11, and 09-2F8 failed to effectively neutralize the aGV vaccine strain (IC50 > 1000 ng/mL) and exhibited highly variable potencies against other strains (**Figure 1b**). MAb 09-1G11 showed the narrowest coverage, failing to neutralize aGV, CTN-1V, and PV (IC50 > 1000 ng/mL) (**Figure 1b**), while 15-2G4-1F2 displayed lower overall neutralizing potency (**Figures 1a, b**). Based on their high neutralization breadth across street isolates and consistent potency across all major vaccine strains, 05-2F10, 06-2A12, and 07-4D8-1C1 were selected as the optimal candidate pool for establishing a universal quantification assay.

### 2.2 Fine mapping of key amino acid residues critical for mAb binding

To delineate the structural epitopes recognized by the three leading candidates (05-2F10, 06-2A12, and 07-4D8-1C1), we executed systematic site-directed mutagenesis targeting previously defined antigenic sites on the RABV G protein: antigenic site I (residues 226–231), site IIa (residues 34–42), site IIb (residues 198–200), site III (residues 330–338), site minor a (residues 342–343), and site G5 (residues 261–264). A total of 64 single-point mutations were introduced into the CVS-N2c background to construct a mutant pseudovirus panel for high-throughput neutralization screening. Of note, mutations at position C228 (C228Y/S/Q/R/W) severely compromised viral infectivity or pseudovirus packaging, precluding their inclusion in subsequent assays.

Neutralization fingerprinting revealed that compared to the parental CVS-N2c reference strain, the sensitivity of mutants G34V, C35W, and T36D to mAb 05-2F10 was drastically reduced by 143-fold, 214-fold, and 177-fold, respectively (**Figure 2a**). Appreciable reductions in sensitivity were also observed for mutants C35S (78-fold), T36S (25-fold), and L38Q (71-fold) (**Figure 2a**). These data conclusively demonstrate that mAb 05-2F10 predominantly targets antigenic site IIa, with G34, C35, and T36 serving as the core contact residues.

**Figure 2:**
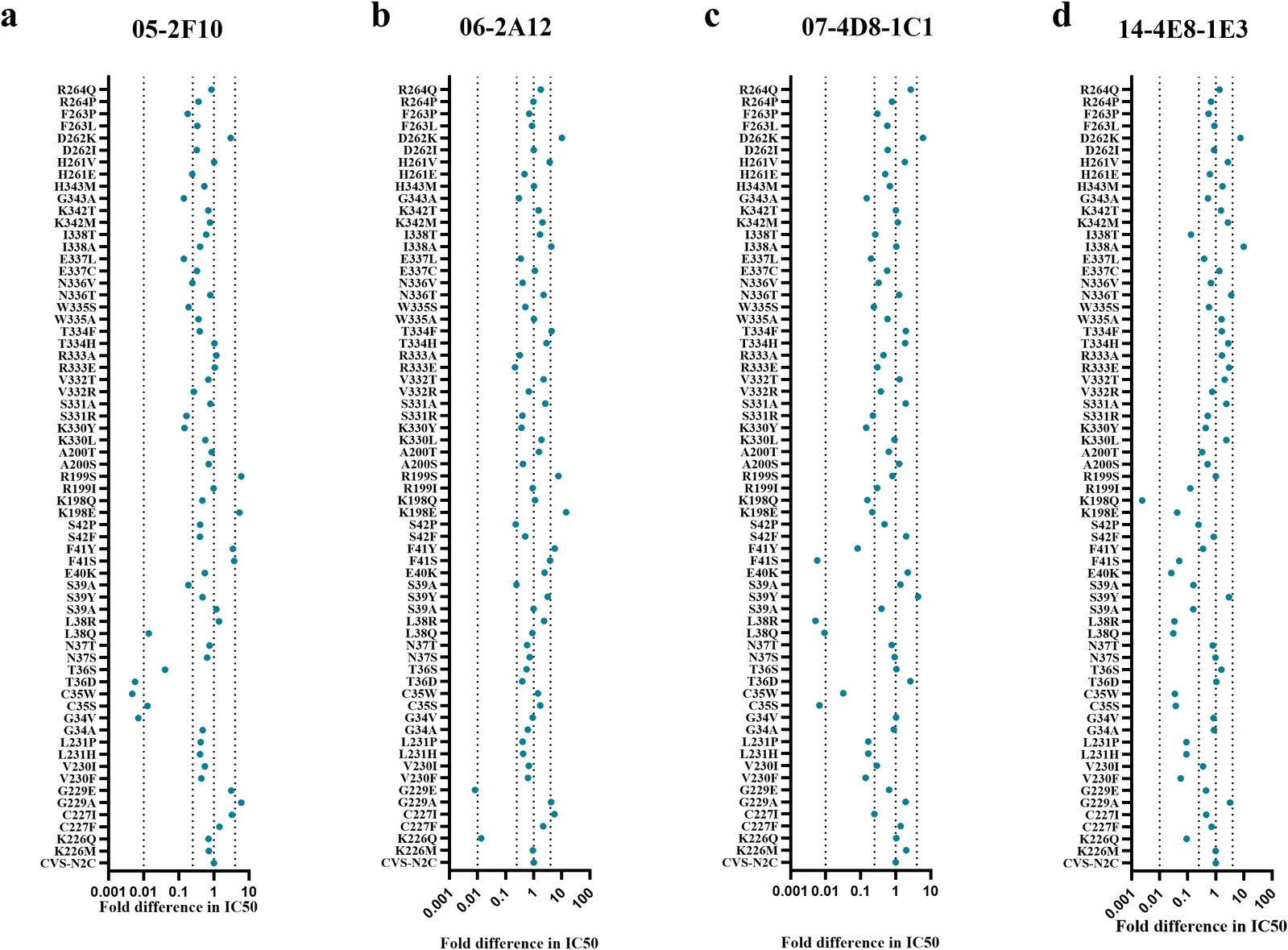
Variations in the neutralization sensitivity of the mutant pseudovirus to four mAbs. Using the CVS-N2C plasmid as a skeleton plasmid, the amino acids in the G protein epitope were mutated one by one, and a pseudovirus was prepared. Changes in neutralizing sensitivity were reflected by the ratio of neutralizing activity of mAbs against mutant pseudoviruses and CVS-N2C strains. The X-axis represents fold-changes in the neutralization sensitivity of mutant strains to mAbs, using CVS-N2C as a reference. The Y-axis represents amino acid mutation sites, which from the bottom to the top are the amino acid sites of epitope I (amino acids 226–231), epitope II (amino acids 34–42 and 198–200), epitope III (amino acids 330–338), epitope a (amino acids 342–343), and epitope G5 (amino acids 261–264). Panels a, b, and c show the changes in neutralization sensitivity of the mutant pseudovirus to mAbs 05-2F10, 07-4D8-1C1, and 06-2A12, respectively.

In contrast, mAb 06-2A12 neutralization was profoundly impacted by mutations within antigenic site I. Specifically, the K226Q and G229E substitutions led to significant 75-fold and 123-fold drops in neutralization sensitivity relative to the wild-type virus, respectively (**Figure 2b**), identifying K226 and G229 as crucial components of the 06-2A12 binding epitope.

For mAb 07-4D8-1C1, single-point mutations at residues C35S, L38Q, L38R, and F41S resulted in exceeding 100-fold decreases in sensitivity, corresponding to reductions of 149-fold, 107-fold, 193-fold, and 172-fold, respectively (**Figure 2c**). Modest resistance was also mediated by C35W (31-fold) and F41Y (12-fold) (**Figure 2c**). These profiles indicate that like 05-2F10, mAb 07-4D8-1C1 targets antigenic site IIa, though it exhibits a distinct dependency on residues C35, L38, and F41.

To visualize the spatial arrangement of these functional epitopes, key contact residues identified via mutagenesis were mapped onto the 3D cryo-EM structure of the prefusion RABV G protein trimer (PDB ID: 7U90). Residues K226 and G229 form a exposed loop region, whereas G34, C35, T36, L38, and F41 cluster within an adjacent surface-accessible helix-turn motif. The distinct spatial distribution of these conserved conformational epitopes explains the non-competitive binding behavior and complementary neutralization profiles observed when pairing these mAbs in our double-antibody sandwich ELISA.

In summary, epitope mapping confirmed that the critical residues for mAbs 05-2F10, 06-2A12, and 07-4D8-1C1 map distinctly to antigenic sites IIa, I, and IIa, respectively.

### 2.3 Development of a double-mAb sandwich ELISA for G protein quantification

To identify optimal antibody pairs for an antigen-quantification assay, the *in vitro* neutralizing activities of all eight mAbs were cross-evaluated against five standard vaccine strains (aGV, CTN-1V, PV, Flury-LEP, and PM) via pseudovirus assays (**Figure 1b**). MAbs 05-2F10 and 07-4D8-1C1 displayed exceptional potency across all five strains, with IC50 values spanning 0.68–5.02 ng/mL and 1.52–7.05 ng/mL, respectively (**Figure 1b**). These were followed by 06-2A12 (IC50: 0.16–539 ng/mL) and 15-2G4-1F2 (IC50: 0.45–700 ng/mL).

Conversely, mAbs 05-2C12, 09-3B10-1G11, and 09-2F8 failed to effectively neutralize the aGV strain (IC50 > 1000 ng/mL) and displayed highly variable potencies against the remaining four vaccine strains, with IC50 values ranging from 4.02 to 163 ng/mL, 2.47 to 28.93 ng/mL, and 3.29 to 33.71 ng/mL, respectively (**Figure 1b**). MAb 09-1G11 exhibited the narrowest coverage, failing to neutralize the aGV, CTN-1V, and PV strains (IC50 > 1000 ng/mL), though it retained strong activity against Flury-LEP (3.22 ng/mL) and PM (2.1 ng/mL) (**Figure 1b**).

Based on the superior broad-spectrum neutralization profiles and the non-competitive epitope characteristics (Site I vs. Site IIa), a complementary antibody matrix was designed: mAbs 07-4D8-1C1 and 06-2A12 were combined in an equal ratio to serve as the solid-phase capture (coating) antibodies, while horseradish peroxidase-conjugated 05-2F10 (05-2F10-HRP) was selected as the fluid-phase detection antibody. Although 07-4D8-1C1 (capture) and 05-2F10 (detection) both recognize antigenic site IIa and share contact residue C35, their distinct fine-epitope footprints permit non-competing engagement. Furthermore, incorporating 06-2A12 (Site I) in the capture dual-antibody cocktail ensures efficient, non-overlapping antigen immobilized on the plate, leaving ample accessible sites for 05-2F10-HRP detection without steric hindrance.

### 2.4 Methodological validation of the double-mAb sandwich ELISA

The analytical performance of the newly developed double-mAb sandwich ELISA was rigorously validated. To assess analytical sensitivity, the optical density at 450 nm (OD450) was plotted against a series of known antigen reference concentrations and fitted using a four-parameter logistic (4PL) regression model (**Figure 3a**). The linear range of the assay was determined to be 0.003125–0.05 IU/mL, with a lower limit of detection (LLOD) established at 0.003125 IU/mL. The derived standard curve equation was Y = 64.81X - 0.08275, achieving an excellent correlation coefficient (R^2^ = 0.9994) (**Figure 3b**).

**Figure 3:**
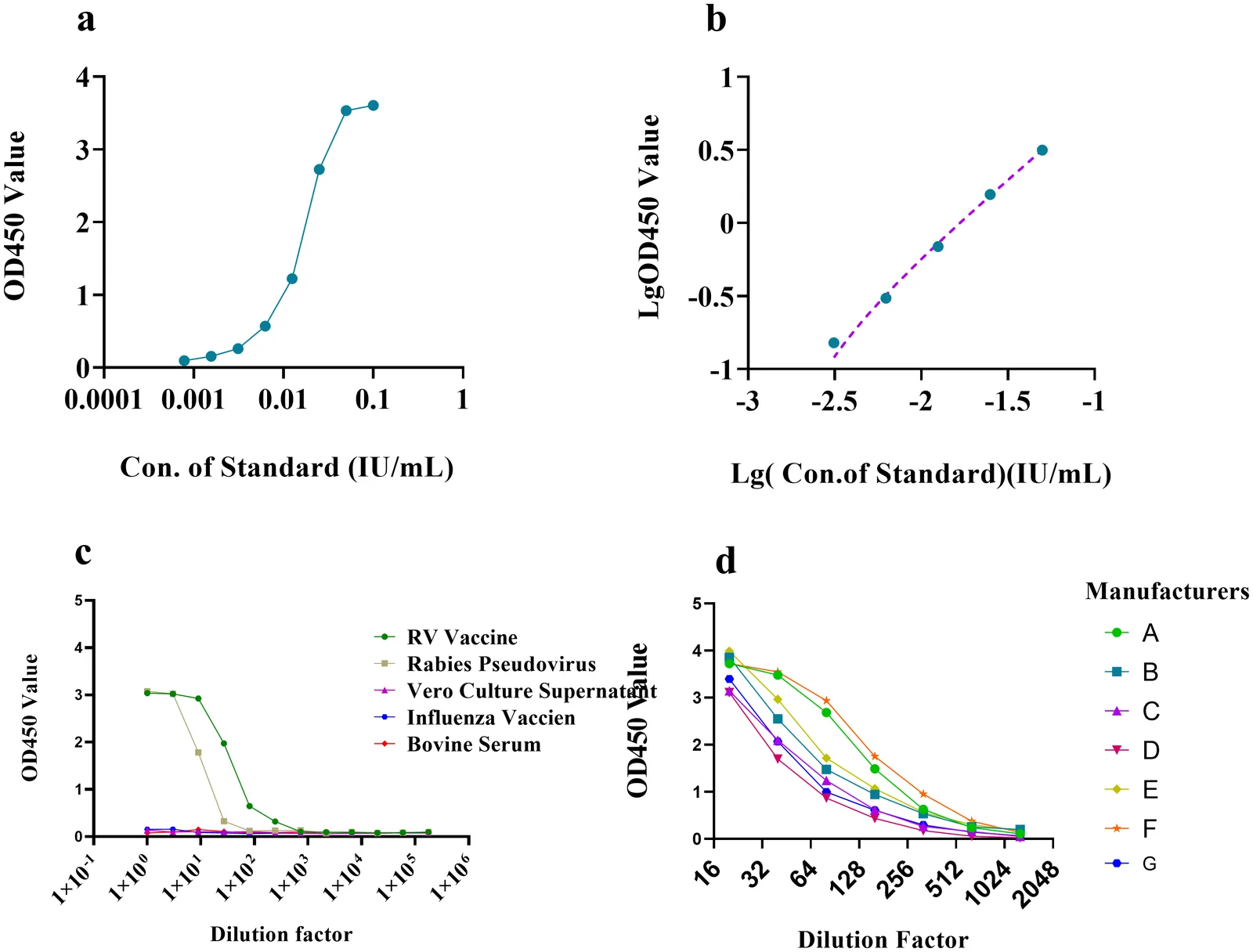
Methodology validation of the newly-developed double antibody sandwich ELISA method. Panels **(a, b)** show the sensitivity test results of the double antibody sandwich ELISA. The antigen standard was diluted from 5 IU/ml to 0.1 IU/ml, then a two-fold gradient dilution was used to determine the absorbance value (OD value) at A450. The antigen reference concentration is used as the abscissa, and the OD value is used as the ordinate. Four parameters are fitted. Panel **(a)** shows the measurements obtained with the antigen standard, with the X-axis representing the antigen standard concentration and the Y-axis representing the OD450 value. Panel **(b)** shows a fitting curve obtained by converting the data from the X and Y axes of panel a logarithmically. Panel **(c)** shows the specificity data obtained for the double antibody sandwich ELISA. The rabies vaccine, influenza vaccine, rabies virus pseudovirus, bovine serum, and Vero cell culture supernatant were included in the analysis. The X-axis displays the sample dilution ratio, and the Y-axis displays the OD450 value. Panel **(d)** shows the results obtained for human rabies vaccines from seven different manufacturers by double antibody sandwich ELISA. Human rabies vaccines from seven different manufacturers were diluted twice and 100 µL was used to detect the glycoprotein content. The X-axis displays the sample dilution factor, and the Y-axis displays the OD450 value.

The specificity of the assay was verified by testing commercial human rabies vaccines, rabies pseudoviruses, commercial influenza vaccines, Vero cell culture supernatants, and fetal bovine serum. The assay generated robust, dose-dependent positive signals only for rabies vaccines and rabies pseudoviruses (**Figure 3c**), whereas influenza vaccines, cell culture supernatants, and bovine serum samples yielded flat baseline negative readings, demonstrating high specificity and negligible matrix interference from non-RABV components.

Assay precision was determined by quantifying high-concentration (0.0125 IU/mL) and low-concentration (0.00625 IU/mL) vaccine standards across 10 replicates per day over a 5-day validation period (**Tables 1**, **2**). Except for a single intra-day CV of 16.40% observed on Day 4 for the low-concentration standard—likely due to increased baseline signal variation near the lower detection boundary—all other intra-day CVs (4.60%–15.00%) and inter-day CVs (13.80% and 14.20%) remained within the 15% acceptance criterion, demonstrating reliable assay reproducibility.

**Table 1:** Inter-day and intra-day precision of the high concentration standard.

Experiment day	OD450 value										Intra-day precision		
	1	2	3	4	5	6	7	8	9	10	Mean	SD	CV
Day1	0.808	0.8	0.821	0.816	0.929	0.927	0.887	0.86	0.862	0.842	0.855	0.047	5.50%
Day2	0.713	0.712	0.745	0.744	0.748	0.82	0.809	0.758	0.739	0.832	0.762	0.043	5.70%
Day3	0.711	0.737	0.725	0.727	0.729	0.793	0.794	0.74	0.754	0.81	0.752	0.035	4.60%
Day4	1.005	0.923	0.868	1.063	1.09	1.089	0.968	0.999	0.922	1.011	0.994	0.075	7.50%
Day5	1.002	0.919	0.864	1.062	1.087	1.088	0.965	0.995	0.918	1.01	0.991	0.076	7.60%
Inter-day precision											0.871	0.12	13.80%

**Table 2:** Inter-day and intra-day precision of the low concentration standard

Experiment day	OD450 value										Intra-day precision		
	1	2	3	4	5	6	7	8	9	10	Mean	SD	CV
Day1	0.278	0.335	0.352	0.359	0.375	0.384	0.365	0.359	0.299	0.336	0.344	0.033	9.70%
Day2	0.336	0.371	0.391	0.405	0.444	0.394	0.319	0.391	0.334	0.365	0.375	0.038	10.10%
Day3	0.321	0.359	0.381	0.392	0.434	0.383	0.314	0.383	0.326	0.354	0.365	0.037	10.30%
Day4	0.337	0.246	0.325	0.394	0.261	0.254	0.301	0.349	0.256	0.287	0.301	0.049	16.40%
Day5	0.337	0.246	0.325	0.394	0.301	0.349	0.256	0.287	0.366	0.354	0.322	0.048	15.00%
Inter-day precision											0.341	0.049	14.20%

Analytical accuracy was evaluated by recovering target concentrations from diluted vaccine standards. The mean relative deviations across concentrations were within 15%, with individual measurement deviations ranging from 3% to 15.2% (**Table 3**), fulfilling standard regulatory accuracy requirements.

**Table 3:** Analysis of the accuracy of the data.

No.	High concentration standard (0.0125IU/mL)			Low concentration standard (0.00625IU/mL)		
	Measured value (IU/mL)	Theoretical value (IU/mL)	Measurement deviation (%)	Measured value (IU/mL)	Theoretical value (IU/mL)	Measurement deviation (%)
1	0.0144	0.0125	15.2	0.00609	0.00625	3
2	0.0129	0.0125	3	0.00691	0.00625	11
3	0.0131	0.0125	5	0.00668	0.00625	7

Finally, the assay’s matrix tolerance and field applicability were examined using commercial human rabies vaccines sourced from seven distinct manufacturers (**Figure 3d**). The ELISA successfully quantified the G protein content across all formulations in a highly uniform, dose-dependent manner, showing minimal sensitivity variations despite the differences in vaccine strains and host cell substrates utilized by individual manufacturers.

### 2.5 Correlation between *in vitro* antigenic content and *in vivo* potency

To determine whether this optimized *in vitro* ELISA could reliably predict *in vivo* biological potency and serve as a viable alternative to animal-based assays, inactivated commercial vaccine bulks were subjected to accelerated thermal degradation at 50 °C for 0, 1, 2, and 4 hours to induce progressive antigen denaturation. The relative antigen content quantified by the sandwich ELISA decreased linearly over time. Linear regression analysis yielded a degradation slope of -0.2735 (Y-intercept = 0.843, R^2^ = 0.81), representing an average loss rate of 27.13% of baseline potency per hour at 50 °C.

In parallel, these heat-stressed vaccine lots were evaluated using the classical *in vivo* NIH mouse potency assay to determine protection endpoints. As expected, after challenge with the RABV CVS strain, mouse mortality rates showed a clear dose-dependent and heat-treatment-dependent pattern: higher vaccine dilutions and longer thermal stress durations (e.g., 4 h at 50 °C) resulted in significantly increased mortality rates (**Figure 4c**), confirming the progressive loss of protective efficacy. Protection endpoints were calculated via the Reed-Muench formula, and mouse serum samples were collected 7 days post-boost to measure virus-neutralizing titers. Linear regression analysis correlating the log-transformed *in vitro* ELISA antigen recovery against the *in vivo* NIH potency values yielded a high correlation coefficient (R^2^ = 0.91) (**Figure 4d**). Similarly, the *in vivo* protection data correlated strongly with serum antibody titers (R^2^ = 0.72) (**Figure 4e**). These data verify that the double-mAb sandwich ELISA serves as a highly sensitive surrogate indicator of vaccine stability and potency, fully aligning with the 3R principles (Replacement, Reduction, and Refinement) and ICH guidelines for minimizing laboratory animal consumption.

**Figure 4:**
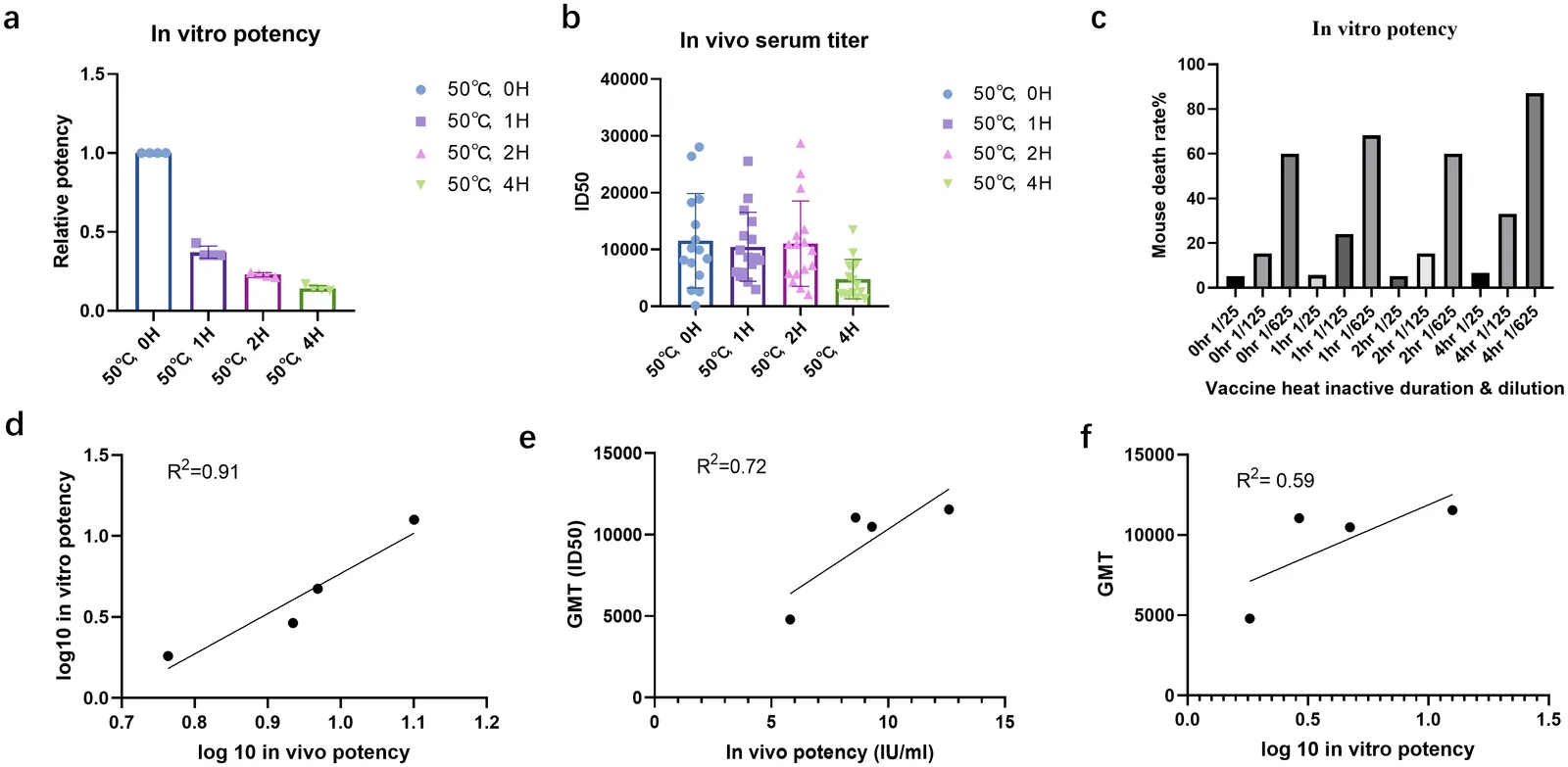
Correlations between *in vivo* potency and *in vitro* potency results for rabies vaccine. Panel **(a)** shows *in vitro* potency tested by ELISA. Each column represents the mean of four replicates. The results plotted are from samples subjected to 50 °C heat-accelerated destruction for different intervals: 0 h, 1 h, 2h and 4h. Panel **(b)** shows antibody titer against CVS strain after two does vaccine administration. Each column represents the mean of 16 mice. Panel **(c)** shows mouse death rate after vaccine administration the challenged with rabies CVS strain. Panel **(d)** shows correlations between *in vivo* potency obtained by NIH test and *in vitro* potency obtained by ELISA. Panel **(e)** shows correlations between *in vivo* potency obtained by NIH test and serum antibody titer. Panel **(f)** shows correlations between *in vitro* potency obtained by ELISA and serum antibody titer. Statistical analyses were performed using GraphPad Prism 8.

## 3 Discussion

Despite the annual administration of rabies PEP to over 15 million individuals globally, fewer than 10% of patients sustaining high-risk Category III exposures actually receive essential RIG treatment. This critical therapeutic gap is driven primarily by the prohibitive manufacturing costs of blood-derived immunoglobulins, severe global supply deficits, and the inherent risk of batch-to-batch variations in serum derivatives (15). Currently, the most viable strategy to replace blood-derived RIG is the development of recombinant monoclonal antibody cocktails. Regulatory frameworks dictate that such cocktails comprise at least two non-competing mAbs targeting highly conserved epitopes on the viral G protein to minimize the risk of neutralization escape among diverse geographic street strains (16, 17).

In this study, we characterized three human-mouse chimeric mAbs (05-2F10, 06-2A12, and 07-4D8-1C1) displaying exceptional neutralization potency and breadth. Importantly, mAb 06-2A12 alone successfully neutralized a diverse panel of 58 regional street virus isolates at concentrations well below 1000 ng/mL. Epitope mapping identified antigenic site I as the target of 06-2A12, with K226 and G229 acting as critical contact residues. Comprehensive sequence alignment data from Wang et al. (18) involving 2890 circulating street isolates showed that positions K226 and G229 exhibit high conservation, with polymorphic stability rates of 99.96% and 100%, respectively. In contrast, mAbs 05-2F10 and 07-4D8-1C1 target distinct patches within antigenic site IIa. Residues G34, C35, and T36 are essential for 05-2F10 binding, while C35, L38, and F41 anchor 07-4D8-1C1. Across the same 2890 street isolates, these positions display conservation rates of 99.90% (G34), 99.97% (C35), 83.22% (T36), 100% (L38), and 99.72% (F41). Although residue T36 exhibits a lower conservation rate (83.22%) compared to G34 and C35 (>99.9%), epitope mapping indicates that binding is predominantly stabilized by the G34–C35 core motif. Nevertheless, potential minor binding variations at T36 should be taken into account when evaluating vaccines derived from rare T36-variant isolates. Because neutralization breadth depends fundamentally on epitope conservation, these high alignment frequencies explain the broad-spectrum efficacy observed for these antibodies and highlight mAb 06-2A12 as a foundational component for future PEP antibody cocktails.

Concurrently, substantial global research has focused on developing *in vitro* immunoassay surrogates to phase out the highly variable animal-based NIH vaccine potency test (19). Double-antibody sandwich ELISAs represent the apex of these efforts due to their superior analytical sensitivity, robust specificity, rapid throughput, and operational safety, making them prime candidates for integration into standard regulatory pipelines (20–24). Because the native, trimeric conformation of the G protein represents the principal protective antigen capable of eliciting protective VNAs, quantifying conformationally intact G protein serves as a direct, reliable surrogate for vaccine efficacy.

However, a major hurdle in standardizing rabies vaccine ELISAs stems from the structural heterogeneity across distinct seed strains (e.g., PM, CTN-1V, PV) and cell substrates (e.g., Vero cells, human diploid cells) used by various global manufacturers. To bridge this gap, we strategically combined mAbs 06-2A12 and 07-4D8-1C1 as the solid-phase capture matrix and paired them with a highly compatible 05-2F10-HRP detection conjugate.

Translating an *in vitro* assay to official regulatory adoption requires adherence to international standards, such as WHO recommendations (TRS No. 941, No. 1012). While single-laboratory validation provides essential proof-of-concept, it is insufficient to establish formal regulatory interchangeability. Formal adoption requires multi-center collaborative studies across independent laboratories using WHO Reference Standards to confirm inter-laboratory reproducibility. Implementing this assay offers substantial 3R benefits. A standard NIH test consumes 100–160 mice per batch. Adopting this high-throughput ELISA nationally in major producing countries like China could save an estimated 50,000 to 80,000 mice annually, while cutting testing time from weeks to hours and eliminating animal suffering.

Despite its strong performance and high correlation with the *in vivo* NIH assay, several limitations of the current study must be acknowledged. First, the assay’s quantitative accuracy relies heavily on the structural integrity and conservation of two specific neutralizing epitopes (Site I and Site IIa). Natural antigenic variations or amino acid substitutions within these epitopes across non-standard or newly emerging vaccine strains may alter monoclonal antibody binding affinity, potentially resulting in under- or over-quantification of the native G protein. Second, this study focused primarily on bulk and unadjuvanted vaccine formulations; further evaluation is required to assess matrix interference or antigen recovery efficiency in adjuvanted finished products, where antigen desorption protocols may need optimization. Third, while *in vitro* G protein content correlates well with mouse challenge protection, uncertainties remain regarding how consistency in ELISA-derived potency metrics translates directly to WHO-recommended *in vivo* immunogenicity thresholds in humans or animal models. Finally, to transition this candidate ELISA from a promising in-process monitoring tool to an internationally accepted batch-release method, comprehensive multi-center cross-validation studies utilizing WHO-recognized International Reference Standards will be essential to establish regulatory equivalence and global applicability.

While the primary application of this double-mAb sandwich ELISA is in-process quality control and batch release, its potential integration into regulatory frameworks to replace the traditional NIH potency assay creates an obligation to address antigen surveillance and field variant detection. Fine-mapping analysis revealed that position T36 (83.22% conserved across 2,890 street isolates) serves as a core contact residue for the sole detection antibody (05-2F10). Naturally occurring T36 variants, while likely retaining G-protein structural integrity and biological function, may demonstrate reduced detection sensitivity in this ELISA system. To mitigate biosafety risks and prevent potential detection evasion by natural field isolates, we recommend that regulatory frameworks adopting this assay mandate routine pseudovirus testing with position-T36 variants (such as T36S, T36D, and newly reported clinical polymorphisms) as an essential component of standard quality-control protocols. This proactive surveillance approach aligns with international regulatory frameworks for monoclonal antibody-based diagnostics and supports ongoing vaccine safety monitoring.

Methodological validation confirmed that this optimized formulation possesses an analytical linear range of 0.003125–0.05 IU/mL, with a fitting linearity (R^2^) exceeding 0.99. The assay proved entirely insensitive to non-specific matrices, including heterologous influenza vaccines, bovine serum, and uninfected cell supernatants. Both intra- and inter-day precision and accuracy parameters comfortably surpassed standard clinical validation criteria (CV% and deviation < 15%). Most importantly, when deployed against commercial vaccine lots from seven different manufacturers, the assay generated highly uniform, dose-dependent quantification profiles. This universal compatibility demonstrates that our ELISA framework can successfully streamline in-process quality control and accelerate routine potency evaluation during rabies vaccine manufacturing. While the current agreement is robust enough to support in-process antigen monitoring and release testing, further international multi-center collaborative studies and expanded lot evaluations will be conducted to establish formal regulatory batch-release equivalence. In addition, while this study established the lower limit of detection (LLOD), a formal lower limit of quantification (LLOQ) and comprehensive robustness evaluations were not fully systematically evaluated. Although not strictly required for the proof-of-concept presented in this initial report, such standard analytical validation steps aligned with ICH Q2(R2) and WHO TRS frameworks are recognized as essential requirements for methods intended to formally replace animal-based regulatory assays like the NIH test. These additional validation metrics will be systematically addressed in our upcoming multi-center collaborative studies to ensure full regulatory compliance and global applicability for batch release. Besides, this study has conducted method applicability validation on commercial rabies vaccines from multiple manufacturers, and the results confirm that this method has favorable detection consistency and applicability across different commercial formulations. Regarding the potential impact of the antigen adsorption state in aluminum-adjuvanted formulations on epitope accessibility, targeted methodological evaluation and optimization can be further carried out in follow-up studies. Aligning with 3R principles, this *in vitro* assay specifically monitors epitope integrity under thermal stress, serving as a complementary tool rather than a direct surrogate for total antigenic mass or overall potency. Because real-world instability may stem from other unmeasured mechanisms such as oxidation, deamidation, or aggregation, the current evaluation reflects antigen structural preservation under heat stress rather than a comprehensive measure of total vaccine stability.

## 4 Materials and methods

### 4.1 Monoclonal antibodies, vaccines, and standards

The eight anti-RABV G protein mAbs evaluated in this study (05-2F10, 06-2A12, 07-4D8-1C1, 15-2G4-1F2, 05-2C12, 09-1G11, 09-3B10-1G11, and 09-2F10) were developed and provided by Biotech (Beijing) Pharmaceutical Technology Co., Ltd. The Chinese National Standard for Rabies Vaccine Efficacy and reference influenza vaccines were supplied by the National Institutes for Food and Drug Control (NIFDC, Beijing, China).

### 4.2 Cell culture

Human embryonic kidney 293T cells (ATCC CRL-3216) were maintained according to established protocols (18) in Dulbecco’s Modified Eagle’s Medium (DMEM; HyClone, Logan, UT, USA) supplemented with 10% fetal bovine serum (FBS; PAN-Biotech, Aidenbach, Germany), 20 mM HEPES (Gibco, Grand Island, NY, USA), and 100 U/mL penicillin-streptomycin (Gibco). Cultures were incubated at 37 °C under a humidified 5% CO_2_ atmosphere.

### 4.3 Construction, production, and titration of RABV Pseudoviruses

The G protein coding sequences of 64 distinct RABV strains were utilized, comprising six fixed laboratory/vaccine strains—PM (NCBI Accession: CAI43218.1), PV (P08667.1), CTN-1V (ACR39382.1), aGV (ADM32132.1), Flury-LEP (ADD84785.1), and CVS-N2c (ADJ29911.1)—and 58 regional street virus isolates belonging to major lineages within Lyssavirus Phylogroup I (**Supplementary Table 1**). All viral codons were optimized for mammalian expression systems and synthesized into the pcDNA3.1(+) vector (General Biosystems, Anhui, China).

When 293T helper cells reached 80%–90% confluence, they were transiently transfected using Lipofectamine 3000 (Invitrogen, Carlsbad, CA, USA) according to the manufacturer’s protocol. Briefly, cells were infected with a single-round replication-deficient G-deficient vesicular stomatitis virus backbone packing a luciferase reporter (Delta G-luciferase pseudovirus; Kerafast, Boston, MA, USA) diluted to 7.0 x 10^4^ TCID50 in DMEM. Following a 3–5 h incubation at 37 °C, the transfection inoculum was aspirated, cells were washed three times with phosphate-buffered saline (PBS) containing 1% FBS, and replenished with fresh complete DMEM. Viral supernatants were harvested at 24 h intervals over a 48 h period, clarified via centrifugation at 1000 rpm for 10 min at 4 °C, passed through a 0.45μm syringe filter (Millipore, Boston, MA, USA), and stored in aliquots at −80 °C.

For pseudovirus titration, harvested stocks were subjected to 5-fold serial dilutions in 96-well plates. Uninfected 293T cells served as negative cell controls. To eliminate thermal edge effects, peripheral wells were filled with 250 μL of sterile water. Following a 24 h incubation, 100μL of supernatant was removed from each well and replaced with 100μL of a luciferase assay reagent. Following a 2 min incubation in the dark, relative light units (RLUs) were recorded using a microplate luminometer (PerkinElmer, Waltham, MA, USA). Wells registering RLU values 3-fold over the background cell controls were scored as positive. Absolute viral titers were calculated via the Reed-Muench method and expressed as TCID50.

### 4.4 *In vitro* pseudovirus neutralization assay

Chimeric mAbs were prepared at an initial concentration of 1000 ng/mL and subjected to 5-fold serial dilutions in complete DMEM. Standardized pseudovirus stocks adjusted to an optimized target titer were mixed 1:1 (50μL: 50μL) with the antibody dilutions in 96-well plates and incubated at 37 °C with 5% CO_2_ for 1 h to allow immune complex formation. Subsequently, 100μL of a 293T cell suspension 4 x 10^5^ cells/mL was added to each well. To validate batch-to-batch assay performance, a standardized reference batch of chimeric mAb 06-2A12 with a known historical IC_50_ profile was included as an internal control on each 96-well plate.

Following a 24 h incubation, luciferase substrate was added and RLU values were recorded. The neutralization percentage was determined by comparing sample RLU values against cell control (100% inhibition) and virus control (0% inhibition) wells. Neutralization curves were plotted, and IC50 thresholds were calculated using the Reed-Muench method (25).

### 4.5 Construction and profiling of mutant pseudoviruses

The wild-type CVS-N2c G protein expression vector pCMV-CVS-N2c served as the template for site-directed mutagenesis (26). A comprehensive panel of 69 single-point amino acid substitutions was constructed (**Supplementary Table 2**). All mutagenic primers were synthesized and sequence-verified by the Beijing Nuosai Genome Research Center Co., Ltd. (Beijing, China). The packaging, harvesting, titration, and neutralization profiles of the mutant pseudoviruses were evaluated using the identical protocols described for the wild-type virus.

### 4.6 HRP conjugation of monoclonal antibodies

Covalent conjugation of horseradish peroxidase (HRP) to the purified mAb 05-2F10 was performed by Beijing Wantai Biological Pharmacy Enterprise Co., Ltd., and the stable enzyme-labeled conjugate was stored in glycerol aliquots at −20 °C.

### 4.7 Standard protocol for the double-mAb sandwich ELISA

Purified mAbs 07-4D8-1C1 and 06-2A12 were blended in an equal protein mass ratio (1:1) in carbonate coating buffer (0.05 M, pH 9.6) to a final concentration of 2mg/mL. Microtiter plates were coated with 100 μL/well of this mixture and incubated overnight at 4 °C. The wells were then washed three times with PBS containing 0.05% Tween-20 (PBST) and blocked with 200μL/well of a blocking buffer at 37 °C for 2–4 h before drying overnight. Antigen standards and vaccine samples were subjected to 2-fold serial dilutions, added at 100μL/well, and incubated at 37 °C for 30 min. Following five washes with PBST, 100μL/well of diluted 05-2F10-HRP conjugate 2 μg/mL) was added and incubated at 37 °C for 30 min. After an additional five washes, color development was initiated by adding 100 μL of tetramethylbenzidine (TMB) substrate (Solutions A and B mix) for 15 min at room temperature before quenching with a stop solution. The OD450 values were determined using a microplate reader.

### 4.8 Validation parameters of the ELISA method

- Specificity: Evaluated by analyzing human rabies vaccines and rabies pseudoviruses alongside heterologous matrices, including commercial influenza vaccines, uninfected Vero cell culture supernatants, and raw fetal bovine serum.
- Sensitivity: Reference standards were prepared from 5 IU/mL down to 0.1 IU/mL via a 2-fold gradient. Absorbance data were parsed using 4PL regression fitting. A log-log transformation was applied to define the baseline linear equation and determine the R^2^ value.
- Precision: Replicate validation sets containing known high-concentration (0.0125 IU/mL) and low-concentration (0.00625 IU/mL) reference controls were evaluated with 10 independent replicates per day over 5 consecutive days to calculate intra-day and inter-day coefficients of variation (CV%).
- Accuracy: Evaluated by testing low (0.00625 IU/mL) and high (0.0125 IU/mL) target controls in triplicate. Accuracy was measured as percent recovery error: measurement deviation = [(measured value - theoretical value)/theoretical value] × 100%.
- Applicability: Comparative performance testing was completed using a commercial glycoprotein determination kit (W and N Company) alongside vaccine formulations from seven different commercial manufacturers.

### 4.9 *In vivo* vs. *in vitro* potency correlation testing

Bulk inactivated rabies vaccine lots were exposed to thermal stress at 50 °C for 0, 1, 2, and 4 h to induce structural degradation. The biological potency of heat-stressed vaccine lots were determined using the active immunization-challenge NIH test following Chinese Pharmacopoeia and WHO guidelines. Briefly, four-week-old female Kunming (KM) mice were immunized intraperitoneally on days 0 and 7 with serial dilutions of test vaccines or the National Reference Standard. On day 14, all mice were challenged intracerebrally with 50 LD_50_ of the CVS-11 strain. Survival was monitored daily for 14 days post-challenge. Relative vaccine potency was calculated from survival data using the Reed-Muench formula in comparison with the National Reference Standard.

For parallel *in vitro* assay verification, the identical vaccine lots were diluted across an 8-point matrix (12 down to 0.09375 IU/mL) in 0.5% BSA-PBST, processed via the double-mAb sandwich ELISA protocol, and detected using 05-2F10-HRP (2 μg/mL). The correlation between the log-transformed *in vitro* antigen recoveries and *in vivo* protection indices was parsed using linear regression analysis in GraphPad Prism 8.

### 4.10 Institutional DURC compliance

In accordance with institutional policies and national guidelines on Dual Use Research of Concern (DURC), a formal biosafety assessment was conducted prior to study initiation and manuscript submission. The study was determined to involve non-replicating pseudotyped viral vectors and diagnostic antibody characterization, posing no restricted dual-use risks, and all data dissemination procedures complied with regulatory transparency and biosafety standards.

## Data Availability

The raw data supporting the conclusions of this article will be made available by the authors, without undue reservation.
