# Supplementary material for: A novel double-mAb sandwich ELISA utilizing broad-spectrum neutralizing antibodies as an in vitro alternative to the rabies vaccine NIH potency assay
Source: Front Immunol. 2026 Oct 7;17:1960986. doi: 10.3389/fimmu.2026.1960986 (PMC13683624; doi:10.3389/fimmu.2026.1960986)
Supplement: Supplementary file 1 [file Supplementaryfile1.docx]

Supplemental Figure1 Inhibition rate curves of monoclonal antibodies

Supplemental Figure2 Bland-Altman analysis of in vitro potency and in vivo potency
