# Supplementary material for: A novel double-mAb sandwich ELISA utilizing broad-spectrum neutralizing antibodies as an in vitro alternative to the rabies vaccine NIH potency assay
Source: Front Immunol. 2026 Oct 7;17:1960986. doi: 10.3389/fimmu.2026.1960986 (PMC13683624; doi:10.3389/fimmu.2026.1960986)
Supplement: Supplementary file 2 [file Supplementaryfile2.docx]

Supplementary Table 1 NCBI accession numbers of the 58 street virus strains

| **Protein Accession Number** | |
| --- | --- |
| AAX85993.1 | AGN94274.1 |
| ABX46663.1 | AGN94294.1 |
| ABZ81200.1 | AGN94375.1 |
| ACG63374.1 | AGN94476.1 |
| ACR50725.1 | AGN94477.1 |
| ACR50728.1 | AGN94515.1 |
| ACR50734.1 | AGN94535.1 |
| ACR50740.1 | AGN94548.1 |
| ADF59197.1 | AGZ88022.1 |
| AEZ55919.1 | AIL01014.1 |
| AEZ55925.1 | AIL01029.1 |
| AEZ55929.1 | AIL01032.1 |
| AEZ55930.1 | AIL01033.1 |
| AFN24083.1 | AIL01048.1 |
| AFN24303.1 | AIT92127.1 |
| AFN24458.1 | AJF83755.1 |
| AFN24522.1 | AKN89648.1 |
| AFN24537.1 | AKN89653.1 |
| AGC12020.1 | AKN89668.1 |
| AGC12029.1 | AKN89673.1 |
| AGC12030.1 | AKN89683.1 |
| AGE31941.1 | ALW83587.1 |
| AGE31951.1 | AMM70627.1 |
| AGN94084.1 | AMR71127.1 |
| AGN94120.1 | APD76994.1 |
| AGN94136.1 | APD77159.1 |
| AGN94215.1 | ASU55969.1 |
| BAN51805.1 | ASV64872.1 |
| BAN51841.1 | BAN14154.1 |

Supplementary Table 2 Single amino acid point mutation primers used in this study

| **No.** | **Site** | **Primer-F（5’-3’）** |
| --- | --- | --- |
| 1 | K226M | AAAAGGAGCATGCAGGCTCATGTTATGTGGAGTTCTTGGAC |
| 2 | K226Q | AAAAGGAGCATGCAGGCTCCAGTTATGTGGAGTTCTTGGAC |
| 3 | C227F | TAAAAGGAGCATGCAGGCTCAAGTTTTGTGGAGTTCTTGGACTTAGACT |
| 4 | C227I | AGTCTAAGTCCAAGAACTCCACATATCTTGAGCCTGCATGCTCCTTTTA |
| 5 | C228W | AAGGAGCATGCAGGCTCAAGTTATGGGGAGTTCTTGGACTTAGACTTAT |
| 6 | C228S | AAGGAGCATGCAGGCTCAAGTTATCTGGAGTTCTTGGACTTAGACTTAT |
| 7 | C228Y | AAGGAGCATGCAGGCTCAAGTTATATGGAGTTCTTGGACTTAGACTTAT |
| 8 | C228R | AAGGAGCATGCAGGCTCAAGTTACGTGGAGTTCTTGGACTTAGACTTAT |
| 9 | C228G | AAGGAGCATGCAGGCTCAAGTTAGGTGGAGTTCTTGGACTTAGACTTAT |
| 10 | G229A | GAGCATGCAGGCTCAAGTTATGTGCAGTTCTTGGACTTAGACTTATGGA |
| 11 | G229E | GAGCATGCAGGCTCAAGTTATGTGAAGTTCTTGGACTTAGACTTATGGA |
| 12 | V230F | TGCAGGCTCAAGTTATGTGGATTTCTTGGACTTAGACTTATGGAT |
| 13 | V230I | TGCAGGCTCAAGTTATGTGGAATTCTTGGACTTAGACTTATGGAT |
| 14 | L231H | AGGCTCAAGTTATGTGGAGTTCATGGACTTAGACTTATGGATGGA |
| 15 | L231P | AGGCTCAAGTTATGTGGAGTTCCTGGACTTAGACTTATGGATGGA |
| 16 | G34A | AATAACCTGGTTGTGGAGGATGAAGCATGTACCAACCTGTCCGAGTTCTCC |
| 17 | G34V | AATAACCTGGTTGTGGAGGATGAAGTATGTACCAACCTGTCCGAGTTCTCC |
| 18 | C35S | AACCTGGTTGTGGAGGATGAAGGAAGTACCAACCTGTCCGAGTTCTCCTAC |
| 19 | C35W | AACCTGGTTGTGGAGGATGAAGGATGGACCAACCTGTCCGAGTTCTCCTAC |
| 20 | T36D | TTGTGGAGGATGAAGGATGTGACAACCTGTCCGAGTTCTCCTA |
| 21 | T36S | TTGTGGAGGATGAAGGATGTTCCAACCTGTCCGAGTTCTCCTA |
| 22 | N37S | TTGTGGAGGATGAAGGATGTACCAGCCTGTCCGAGTTCTCCTACATGGA |
| 23 | N37T | TTGTGGAGGATGAAGGATGTACCACCCTGTCCGAGTTCTCCTACATGGA |
| 24 | L38Q | AGTTCCATGTAGGAGAACTCGGACGGGTTGGTACATCCTTCATCCTCCA |
| 25 | L38R | TGGAGGATGAAGGATGTACCAACCGGTCCGAGTTCTCCTACATGGAACT |
| 26 | S39A | AGGATGAAGGATGTACCAACCTGGCCGAGTTCTCCTACATGGAACTCAA |
| 27 | S39Y | AGGATGAAGGATGTACCAACCTGTACGAGTTCTCCTACATGGAACTCAA |
| 28 | E40A | ATGAAGGATGTACCAACCTGTCCGCGTTCTCCTACATGGAACTCAAAGT |
| 29 | E40K | ATGAAGGATGTACCAACCTGTCCAAGTTCTCCTACATGGAACTCAAAGT |
| 30 | F41S | AAGGATGTACCAACCTGTCCGAGTCCTCCTACATGGAACTCAAAGTGGG |
| 31 | F41Y | AAGGATGTACCAACCTGTCCGAGTACTCCTACATGGAACTCAAAGTGGG |
| 32 | S42F | ATGTACCAACCTGTCCGAGTTCTTCTACATGGAACTCAAAGTGGGAT |
| 33 | S42P | ATGTACCAACCTGTCCGAGTTCCCCTACATGGAACTCAAAGTGGGAT |
| 34 | K198E | TGACATTTTTACCAATAGCAGAGGGGAGAGAGCATCCAACGGGAACAAGACTT |
| 35 | K198Q | TGACATTTTTACCAATAGCAGAGGGCAGAGAGCATCCAACGGGAACAAGACTT |
| 36 | R199I | ATTTTTACCAATAGCAGAGGGAAGATAGCATCCAACGGGAACAAGACTTGC |
| 37 | R199S | ATTTTTACCAATAGCAGAGGGAAGAGCGCATCCAACGGGAACAAGACTTGC |
| 38 | A200S | TTTACCAATAGCAGAGGGAAGAGATCATCCAACGGGAACAAGACTTGCGGC |
| 39 | A200T | TTTACCAATAGCAGAGGGAAGAGAACATCCAACGGGAACAAGACTTGCGGC |
| 40 | K330L | TTGATGGAGGCTGATGCTCACTACCTGTCAGTCCGGACCTGGAATGAGATC |
| 41 | K330Y | TTGATGGAGGCTGATGCTCACTACTACTCAGTCCGGACCTGGAATGAGATC |
| 42 | S331R | TGGAGGCTGATGCTCACTACAAGACAGTCCGGACCTGGAATGAGATCAT |
| 43 | S331A | TGGAGGCTGATGCTCACTACAAGGCAGTCCGGACCTGGAATGAGATCAT |
| 44 | V332R | AGGCTGATGCTCACTACAAGTCACGCCGGACCTGGAATGAGATCATCCC |
| 45 | V332T | AGGCTGATGCTCACTACAAGTCAACCCGGACCTGGAATGAGATCATCCC |
| 46 | R333E | CTGATGCTCACTACAAGTCAGTCGAGACCTGGAATGAGATCATCCCCTC |
| 47 | R333A | CTGATGCTCACTACAAGTCAGTCGCGACCTGGAATGAGATCATCCCCTC |
| 48 | T334H | ATGCTCACTACAAGTCAGTCCGGCACTGGAATGAGATCATCCCCTCAAA |
| 49 | T334F | ATGCTCACTACAAGTCAGTCCGGTTCTGGAATGAGATCATCCCCTCAAA |
| 50 | W335A | CTCACTACAAGTCAGTCCGGACCGCGAATGAGATCATCCCCTCAAAAGG |
| 51 | W335S | CTCACTACAAGTCAGTCCGGACCTCGAATGAGATCATCCCCTCAAAAGG |
| 52 | N336T | ACTACAAGTCAGTCCGGACCTGGACTGAGATCATCCCCTCAAAAGGGTG |
| 53 | N336V | ACTACAAGTCAGTCCGGACCTGGGTTGAGATCATCCCCTCAAAAGGGTG |
| 54 | E337C | ACAAGTCAGTCCGGACCTGGAATTGTATCATCCCCTCAAAAGGGTGTTT |
| 55 | E337L | ACAAGTCAGTCCGGACCTGGAATCTGATCATCCCCTCAAAAGGGTGTTT |
| 56 | I338A | AGTCAGTCCGGACCTGGAATGAGGCCATCCCCTCAAAAGGGTGTTTGAA |
| 57 | I338T | AGTCAGTCCGGACCTGGAATGAGACCATCCCCTCAAAAGGGTGTTTGAA |
| 58 | K342M | CCTGGAATGAGATCATCCCCTCAATGGGGTGTTTGAAAGTTGGAGGAAG |
| 59 | K342T | CCTGGAATGAGATCATCCCCTCAACAGGGTGTTTGAAAGTTGGAGGAAG |
| 60 | G343A | GGAATGAGATCATCCCCTCAAAAGCGTGTTTGAAAGTTGGAGGAAGGTG |
| 61 | G343M | GGAATGAGATCATCCCCTCAAAAATGTGTTTGAAAGTTGGAGGAAGGTG |
| 62 | H261E | CTCCAGATCAGTTGGTGAATTTGCAAGACTTTCGCTCAGACGAGATTGA |
| 63 | H261V | CTCCAGATCAGTTGGTGAATTTGGTCGACTTTCGCTCAGACGAGATTGA |
| 64 | D262I | CAGATCAGTTGGTGAATTTGCACATCTTTCGCTCAGACGAGATTGAGCAT |
| 65 | D262K | CAGATCAGTTGGTGAATTTGCACAAATTTCGCTCAGACGAGATTGAGCAT |
| 66 | F263L | ATCAGTTGGTGAATTTGCACGACCTTCGCTCAGACGAGATTGAGCATCT |
| 67 | F263P | ATCAGTTGGTGAATTTGCACGACCCTCGCTCAGACGAGATTGAGCATCT |
| 68 | R264Q | AGTTGGTGAATTTGCACGACTTTCAATCAGACGAGATTGAGCATCTCGT |
| 69 | R264P | AGTTGGTGAATTTGCACGACTTTCCCTCAGACGAGATTGAGCATCTCGT |

Supplementary Table 3 Neutralization activity of mAbs against rabies viruses

| Number | 05-2F10 | 05-2C12 | 06-2A12 | 09-1G11 | 09-3B10-1G11 | 09-2F8 | 15-2G5-1F2 | | 07-4D8-1C1 | |
| --- | --- | --- | --- | --- | --- | --- | --- | --- | --- | --- |
| AMM70627.1 | >1000 | 9.367560403 | 2.070976584 | 735.8767826 | 109.6716745 | 164.3173147 | 7.26328021 | | 0.154360651 | |
| AIL01033.1 | 1.220803248 | 257.2414262 | 2.611112712 | 72.25252473 | >1000 | >1000 | 1.996209411 | | 0.240085947 | |
| APD76994.1 | 0.367989201 | 0.22062258 | 0.248417408 | 33.72842224 | 0.232990685 | 0.247821114 | 0.222684985 | | 0.420758197 | |
| AMR71127.1 | 245.5800655 | 157.0825641 | 0.152900336 | 86.94205602 | 224.2630297 | 257.2127567 | 4.57960843 | | 154.5950703 | |
| AJF83755.1 | 2.442876288 | 10.01575974 | 6.026415534 | 66.49615237 | 5.681012121 | 10.04294889 | >1000 | | 0.762224974 | |
| BAN51841.1 | 0.197297597 | 36.7914748 | 0.92009664 | 12.20771106 | 189.5676722 | 336.5567674 | 0.171188542 | | 0.186012572 | |
| ACR50725.1 | 1.633538232 | 659.1249311 | 7.27427741 | 141.124463 | >1000 | >1000 | 2.723381771 | | 0.695675342 | |
| ACR50734.1 | 2.572434075 | 10.13023973 | 5.231055221 | 86.97701115 | 5.116318489 | 16.37309763 | >1000 | | 0.957447462 | |
| AIL01048.1 | 3.606727161 | 384.7643919 | 4.733974856 | 96.88420608 | >1000 | 674.5484296 | 3.385253048 | | 0.828545578 | |
| AGC12029.1 | 20.73075682 | 656.8669848 | 20.66723703 | 337.0133301 | >1000 | 427.8511655 | 9.948557293 | | 13.91970916 | |
| AGC12020.1 | 3.088011306 | >1000 | 4.578170354 | 94.62592342 | 380.7167939 | >1000 | 72.59042237 | | 0.954496646 | |
| AIL01014.1 | 0.843221172 | 305.6835189 | 2.591921072 | 172.6258562 | >1000 | 876.533984 | >1000 | | 131.9620715 | |
| AIL01029.1 | >1000 | 550.2036615 | 2.284318893 | >1000 | 337.2062207 | 438.7818481 | 177.3452262 | | 675.368613 | |
| AGN94375.1 | >1000 | 482.6775202 | 55.65448051 | 771.3257411 | >1000 | >1000 | >1000 | | >1000 | |
| AIL01032.1 | 10.32006629 | 121.6658891 | 11.01112404 | 275.7671483 | >1000 | 891.3183066 | 12.94008297 | | 331.806485 | |
| AGN94136.1 | 0.873054193 | 510.0826802 | 2.006048742 | 9.171743917 | 489.200465 | 885.0207394 | 1.901772231 | | 0.314413758 | |
| AKN89648.1 | 0.859947293 | 302.1037425 | 2.266917142 | 30.23861403 | 711.6058527 | >1000 | 4.61408424 | | 0.490848238 | |
| AKN89653.1 | 4.622305687 | 8.612451816 | 11.03869603 | 3.361801523 | 9.52514312 | 9.830837918 | 5.940528207 | | 2.253693762 | |
| AKN89668.1 | >1000 | 130.5352464 | 1.460902007 | >1000 | 102.6241349 | 62.07888697 | 47.8947151 | | 104.5715714 | |
| AKN89673.1 | 223.9825422 | 0.85656134 | 1.102558343 | 226.5765933 | 0.773754269 | 0.929902166 | 342.294167 | | 0.432299268 | |
| AKN89683.1 | >1000 | >1000 | 9.130407179 | >1000 | >1000 | >1000 | >1000 | | >1000 | |
| ABZ81200.1 | >1000 | >1000 | 2.647910909 | >1000 | >1000 | >1000 | 2.566497362 | | 0.381364274 | |
| AIT92127.1 | >1000 | >1000 | 0.35532696 | >1000 | >1000 | >1000 | 0.260083536 | | 0.064565423 | |
| AGN94120.1 | 637.4881248 | 13.94544116 | 3.239149917 | >1000 | 5.138054088 | 16.52977686 | 4.411297048 | | 109.9377711 | |
| AFN24537.1 | >1000 | >1000 | 143.3087663 | >1000 | >1000 | 366.9849768 | 282.3849501 | | 612.7690499 | |
| ALW83587.1 | 635.0527147 | 1.359749912 | 1.027641158 | 446.8368956 | 3.886789392 | 0.976811303 | 0.709429474 | | 2.207730538 | |
| AGN94274.1 | 1.320708488 | 0.560705802 | 1.241560554 | 62.73085091 | 1.668673215 | 0.779601923 | 0.769121833 | | 2.129227355 | |
| BAN51805.1 | 0.713609708 | 190.3216547 | 1.432924565 | 145.5906352 | >1000 | >1000 | 0.467250204 | | 0.286053029 | |
| ABX46663.1 | 1.174293382 | 0.804286512 | 0.365235796 | 175.2215935 | 0.633848961 | 0.317315574 | 3.213556079 | | 1.091764971 | |
| AGE31951.1 | 0.287507149 | 0.13013384 | 0.064565423 | 54.11695265 | 0.162990451 | 0.271643927 | 0.553774984 | | 0.383878287 | |
| APD77159.1 | 0.371606156 | 1.464059783 | 0.393431614 | 28.65242965 | 0.599298529 | 0.269206822 | 77.23066196 | | 0.097962115 | |
| BAN14154.1 | 0.307436738 | 3.787928518 | 0.982891144 | 7.783373838 | 0.902708622 | 108.1222533 | 3.159317778 | | 1.081427921 | |
| ASV64872.1 | 0.483020581 | 0.268538007 | 0.327796699 | 31.86920112 | 0.264502774 | 0.484787371 | 0.688158706 | | 0.156330449 | |
| ASU55969.1 | >1000 | 0.956234361 | 0.59095566 | >1000 | 0.581893774 | 1.325495057 | >1000 | | 0.596061137 | |
| AGC12030.1 | 882.5471435 | 448.1005339 | 0.281412562 | 649.2436939 | 4.508317736 | 674.1135512 | 0.444091512 | | 533.8621417 | |
| AEZ55925.1 | 17.79394036 | 10.85702506 | 12.52036815 | 3.36356817 | 6.046262592 | 5.632445632 | 8.122255976 | | 4.158572487 | |
| AFN24522.1 | 4.276648193 | 3.253485689 | 3.518585217 | 109.2654386 | 2.333235844 | 1.21800216 | 1.013481541 | | 1.395799499 | |
| AEZ55930.1 | 271.7657131 | 267.7022157 | 137.6192447 | 273.7178433 | >1000 | 25.61351015 | 1.603740069 | | 4.67182459 | |
| AEZ55929.1 | 0.633467942 | 6.164252812 | 2.27692785 | 17.92690334 | 14.22572524 | 9.561821684 | 2.282611384 | | 9.816714595 | |
| AFN24303.1 | 2.389375011 | 176.2376599 | 303.8344194 | 7.985955472 | >1000 | 2.447414611 | 2.539260879 | | 3.859234444 | |
| AFN24458.1 | 2.489203571 | 520.7171517 | 5.258798689 | 94.65372123 | >1000 | >1000 | 5.311250287 | | 0.961363389 | |
| AGN94515.1 | 166.634343 | 4.225358287 | 2.674748278 | >1000 | 1.71582566 | 2.67741875 | 1.478745153 | | 42.49375665 | |
| AGE31941.1 | 0.510803478 | 1.53043294 | 1.226658647 | 38.21831745 | 2.271412547 | 1.43892883 | 1.226542772 | | 0.898461192 | |
| AFN24083.1 | 0.216190219 | 0.171631394 | 0.353598349 | 5.460111252 | 0.165076341 | 0.236783729 | 0.171468439 | | 0.110744864 | |
| AGN94535.1 | 0.63776796 | 0.739881145 | 0.679439994 | 63.14754939 | 0.786828136 | 1.068522286 | >1000 | | 0.752434776 | |
| ACG63374.1 | 3.559205862 | 11.34428588 | 22.05914662 | 101.5216282 | 9.921719366 | 11.31401738 | 756.0173788 | | 1.2477716 | |
| ACR50728.1 | 2.63952709 | 2.542812249 | 2.205585199 | 109.4086675 | 4.091359437 | 4.537246306 | 2.828745399 | | 2.039656805 | |
| ACR50740.1 | 0.404355484 | 0.255343958 | 0.690812569 | 69.11571903 | 0.559114953 | 1.173068609 | 790.0253643 | | 0.781883851 | |
| AAX85993.1 | 0.880704394 | 1.420506004 | 0.90615195 | 63.1468868 | 1.553206698 | 2.423202067 | >1000 | | 1.775520549 | |
| AGZ88022.1 | 1.969635332 | 4.361529962 | 4.821257058 | 151.6549442 | 2.974261929 | 5.109941363 | 3.269982019 | | 1.005808045 | |
| AEZ55919.1 | 0.590770705 | 222.2983464 | 18.54172885 | 28.97516225 | >1000 | >1000 | >1000 | | 0.476059162 | |
| ADF59197.1 | 15.09880926 | >1000 | 12.10074285 | 106.6539913 | 467.5470046 | >1000 | >1000 | | 18.7756851 | |
| AGN94215.1 | 0.905576855 | 1.998450621 | 2.425579969 | 132.0538015 | 1.863393793 | 2.865443408 | 1.916865174 | | 0.886298116 | |
| AGN94084.1 | 0.691368397 | 0.48074479 | 0.367050491 | 116.9636618 | 0.329939959 | 0.585311371 | 0.574770553 | | 0.843945107 | |
| AGN94294.1 | >1000 | 299.953172 | 9.260283336 | >1000 | 782.9602153 | >1000 | 164.1875564 | | 3.402346405 | |
| AGN94548.1 | 234.5604493 | 3.747517001 | 0.346024884 | 15.21215955 | 3.742535457 | 52.568204 | 0.610108422 | | 0.65876452 | |
| AGN94476.1 | 2.47921946 | 588.9014736 | 12.17237625 | 212.2670376 | >1000 | >1000 | 5.48103611 | | 113.5316602 | |
| AGN94477.1 | >1000 | 104.5471833 | 2.734129023 | 902.2756962 | 356.2908241 | 611.9622742 | 73.12623956 | | 2.760870837 | |
| ADJ29911.1 | 3.461320296 | 15.12 | 3.82193537 | 4.011561134 | 6.67 | 146.9680137 | 12.44171369 | | 0.433165416 | |
| aGV | 1.33 | >1000 | 32.09 | >1000 | >1000 | >1000 | | 84.03 | | 5.23 |
| CTN-1V | 0.91 | 41.58 | 1.92 | >1000 | 2.76 | 0.56 | | 259.58 | | 4.72 |
| Flury-LEP | 1.3 | 4.02 | 3.15 | 3.22 | 2.57 | 3.29 | | 1.09 | | 3.75 |
| PM | 1.54 | 153.02 | 0.16 | 2.1 | 0.3 | 3.58 | | 2.9 | | 5.36 |
| PV | 5.02 | 163.49 | 539.17 | 2000 | 28.93 | 33.71 | | 699.79 | | 7.05 |
